# Barriers and facilitators of HIV vaccine and prevention study participation among Young Black MSM and transwomen in New York City

**DOI:** 10.1371/journal.pone.0181702

**Published:** 2017-07-19

**Authors:** Sharise Richardson, Pich Seekaew, Beryl Koblin, Tasha Vazquez, Vijay Nandi, Hong-Van Tieu

**Affiliations:** 1 University of Miami School of Medicine, Miami, Florida, United States of America; 2 Department of Prevention, Thai Red Cross AIDS Research Center, Bangkok, Thailand; 3 Laboratory of Infectious Disease Prevention, Lindsley F. Kimball Research Institute, New York Blood Center, New York, New York, United States of America; Universidade Estadual de Maringa, BRAZIL

## Abstract

**Background:**

Black men who have sex with men (MSM), and Transwomen (TW) shoulder disproportionate burden of HIV. However, they are unrepresented in HIV vaccine trials. We investigated the perceptions of that factors associated with HIV vaccine trials participation among Black MSM and TW in New York.

**Methods:**

Self-administered online questionnaires were administered to 18–29 years of NYC residents who identified as Black MSM and TW, assessing demographics, awareness and willingness to participate in HIV vaccine trials, barriers and facilitators associated with willingness, and sexual behaviors. Frequency summation was performed to determine barriers and facilitators, and logistic regression analysis was performed to determine factors association with expressed willingness.

**Results:**

Black MSM and TW who reported engaging in risk behaviors had a 61% lower likelihood of participating in HIV vaccine trials when compared to those who did not report engaging in any risk behavior. Facilitators associated with trial participation were: cash compensation, confidentiality regarding participation, public transportation vouchers, gift cards, and food or grocery vouchers as potential facilitators for trial participation. Conversely, fear of side effects from the vaccine, concerns about testing positive on routine HIV testing due to an HIV vaccine, limited knowledge of research trials, and fear of being judged as HIV-positive were perceived as barriers.

**Conclusions:**

These findings provided insights into the considerations and perceptions of Black MSM and TW towards HIV vaccine trials. However, further studies are needed to delineate the complex mechanisms underlying the decision-making process and establish approaches to increase study participation in this population.

## Introduction

Men who have sex with men (MSM) and Transgender Women (TW) have been disproportionately affected by the HIV/AIDS epidemic. In 2014 MSM accounted for 83% of all HIV incidence in men and 67% of all HIV incidence in the U.S. Black MSM (BMSM) comprised the largest number of new diagnoses [1] (CDC 2014). Despite an increase in new diagnoses among overall BMSM, young BMSM (aged 13–24) experienced only a slight decline of 2% in new diagnoses since 2010 [2]. TW have also shouldered a large portion of the HIV burden in the U.S. [3] [4]. In a meta-analysis, Herbst et al found that approximately 27% of TW were HIV-infected at study initiation and 12% self-reported having HIV [4]. Moreover, Black TW had higher rates of HIV infection, with 56% being newly diagnosed and 30% self-reporting being HIV-positive [4].

### Current state of HIV prevention research

Due to the staggering HIV infection rates in BMSM and TW in the US, HIV prevention measures targeted at these populations are important. The use of antiretroviral drugs to prevent HIV acquisition is a key approach. Pre-Exposure Prophylaxis (PrEP) with the use of daily oral emtricitabine /tenofovir disoproxil fumarate has shown to reduce the risk of HIV acquisition by 86% [5]. However, limited long-term safety information, and issues with drug adherence have prompted research looking into alternative oral agents, long-acting injectable formulations and intermittent dosing schedules for oral PrEP [6, 7].

Despite many vaccine trials conducted, six vaccine regimens have made it to clinical efficacy trials [7,8]. The RV144 trial conducted in Thailand was the first to show a reduction in HIV acquisition [9]. Although not demonstrated in the RV144 trial, one potential consequence of receiving vaccines is Vaccine-Induced Sero-Positivity (VISP), which results in false positive to standard HIV serologic tests [10]. This could present several challenges, including disruption of personal relationships and difficulties in obtaining life insurance [10], which could lead to a reluctance to participate in preventive HIV vaccine trials [10, 11].

### Minority participation and population targeted recruitment efforts

Though disproportionately affected by HIV, Blacks have been underrepresented in HIV treatment trials [12]. Insufficient representation of young BMSM and TW in HIV prevention trials may minimize the effort in curtailing HIV epidemic, as the results from such trials would limit the generalizability to these populations. Thus, it is imperative to assess factors that contribute to a low participation in HIV vaccine trial.

We conducted an online survey to assess awareness and perceptions of young BMSM and TW in New York City (NYC) in regards to participating in HIV biomedical intervention research studies.

## Methods

### Recruitment, eligibility, and reimbursement

Eligibility was defined as: self-identify as male, between the ages of 18 and 29, resident in NYC, self-report of anal sex with a man in the past 6 months (insertive and/or receptive), self-identify as Black/African-American, and self-report being HIV-negative. Participants were recruited using banner advertisement placed on several websites (Facebook, Ronaldmatters, Grindr, BGC, and Mused Magazine) frequented by young BMSM and TW living in NYC. Participants were reimbursed with a $30 Amazon.com gift card upon completion of the survey. The study was approved by the New York Blood Center Institutional Review Board (IRB: 543398).

### Survey components

#### Prescreener and main survey questionnaire overview

Participant eligibility and demographics (age, race/ethnicity, gender and sexual identity) were ascertained by an 8-question prescreening questionnaire that was linked to the banner advertisements (S1 File). Participants who were eligible and who indicated interest in participating were automatically redirected to the main study questionnaire on SurveyGizmo (S2 File). The average time to complete the main questionnaire was 21 minutes. No personal identifying information was collected. To preserve the integrity of the questionnaire, responses were limited to one person per IP address. The main study survey consisted of 6 sections: demographics, awareness of HIV prevention approaches, willingness to participate (WTP) in HIV vaccine trials, barriers and facilitators to participating in HIV vaccine trials, and sexual behaviors.

Participant age, race/ethnicity, gender, and sexual identity were asked of participants on the online eligibility prescreening questionnaire. In order to protect participant identification, data on the online prescreening questionnaire including sexual identity and age were not linked with the main questionnaire and thus were not available for analysis.

#### Demographics

The demographics section contained 10 questions including level of education, annual personal income, religion, Hispanic ethnicity, employment, place of birth, usual place of medical care and date of last HIV test.

#### Awareness of HIV prevention approaches

We asked participants if they had ever heard of HIV vaccine research and VISP, participated in HIV vaccine trials, and whether they knew anyone else who has participated in an HIV vaccine trial. We also assessed awareness of PEP and PrEP as well as PrEP trials, use of PEP and PrEP and participation in PrEP trials.

#### Willingness to participate in HIV vcaccine trials

After showing educational information on HIV vaccines trials, participants were asked about their WTP in HIV vaccine trials. Responses were collected on a 4-point Likert scale (not willing, minimally willing, moderately willing, and very willing), and were dichotomized (not willing to participate and willing to participate) for analysis.

#### Barriers and facilitators to participate in HIV vaccine trials

The facilitators were obtained from the questionnaire “Please rate the importance of the following items in terms of what would make your participation in a research trial easier or harder.” The choices were: private transportation to and from appointments, public transportation (bus, metro) vouchers, cash compensation for time, food or grocery vouchers, gift cards, referrals for other community services, condoms and dental dams, hygiene kits (toothbrush, toothpaste, bleach, soaps, shampoo, etc.), clothing, weekday morning study visits, weekday evening study visits, weekend study visits, going to a site close to home, confidentiality regarding participation, other commitments (work, family, etc.), child care or reimbursement for cost of child care.

The barriers were obtained from the questionnaire “What would prevent you from participating in an HIV vaccine trial? (Mark all that apply).” The choices were: access to study site, fear of being judged as HIV positive, fear of being judged of being at risk for HIV infection, fear of being judged for being gay, fear of testing false positive on routine HIV testing, limited knowledge of research trials, fear of needles and injections, fear of side effects from the vaccine, fear of being discriminated against because of race, fear of being discriminated against because of age, nothing: I would be willing to participate.”

#### Sexual behaviors

We asked participants if they ever received or given money, drugs, gifts or services in exchange for sex in the last 12 months, diagnosed with sexually transmitted infections (STIs) (gonorrhea, chlamydia, syphilis) in the past 12 months, and used drugs (injectable drugs, cocaine, methamphetamine/amphetamine) in the past 12 months. Additionally, we asked the participants regarding their sexual position(s) (insertive, receptive) and if they used condoms in the past 6 months

### Statistical analysis

Frequencies of demographic and behavioral characteristic responses were described via univariate logistic regression analyses through TABULATE command in Stata Version 14.0 for Mac (StataCorp 1985–2015, College Station, Texas).

The facilitators and barriers were determined by calculating the sum of frequency of all the choices, and the 5 factors with the highest frequencies were selected.

The primary outcome was WTP in HIV vaccine trials. The outcome was dichotomized into “willing to participate” (participants who responded “moderately willing” and “very willing” to participate in HIV vaccine trials were) and “not willing to participate” (participants who responded “not willing” and “minimally willing” to participate in HIV vaccine trials).

Participants were assigned a Risk Score (RS) of 1 if they had responded yes to one or two of the following five risk behaviors: received money, gifts, drugs for sex; gave money, gifts, or drugs for sex; STIs in past 12 months; any condomless receptive anal intercourse (RAI) in the past 6 months; and any condomless insertive anal intercourse (IAI) in the past 6 months. Those with three or more of the risk behaviors were assigned a RS of 2, the maximum possible RS. Those without any of the risk behaviors were assigned a RS of 0.

Bivariate logistic regression analyses were conducted to determine specific variables that were significantly associated with the outcome (WTP in HIV vaccine trials) using Pearson’s Chi-square test or Fisher’s Exact test among those who reported not having ever participated in HIV vaccine trials in the past. Demographic and behavioral variables that were considered for inclusion were the following: education, employment status, income, place of birth, religion, usual place of medical care, last HIV test, risk behaviors, PrEP usage in the past, PEP usage in the past, and past research participation. Variables with p< 0.05 were considered significantly associated with the outcome and entered into the multivariable logistic regression model. Collinearity between each significant variable was assessed using Pearson’s Chi-square and Fisher’s Exact test, and the Hosmer and Lemeshow’s goodness of fit test was used to evaluate fit of the final model. All statistical analyses were evaluated for statistical significance at α = 0.05, using two-sided tests and performed using Stata Version 14.0 for Mac (StataCorp 1985–2015, College Station, Texas).

## Results

### Population demographics and characteristics

A total of 192 completed the prescreener questionnaire, of whom 45% (N = 85) were between the ages of 18–23, 54.5% (N = 103) were between 24 and 29, and 0.5% (N = 1) was older than 29. 189 participants were enrolled from April to June 2014 with 39.2% identifying as Hispanic (Table 1). Most of the participants were born in the U.S. (N = 172; 91.0%) and identified as Christian (N = 149; 78.8%). Over three-quarters (N = 147; 77.8%) had a college degree or higher, 58.2% (N = 110) of the participants worked full-time, and 33.9% (N = 64) reported having an annual income between $20,000 and $39,999. Most (N = 121; 64%) cited their private doctor as their place for usual medical care, while others reported going to the health department/emergency room (ER) (N = 36; 19.1%) for care.

**Table 1:** Sociodemographics, risk behaviors and past research experience (N = 189).

		N(%)
**Age**		
18–23		85(45.0)
24–29		103(54.5)
30		1(0.5)
**Ethnicity**		
Hispanic African-American		74(39.2)
Non-Hispanic African-American		115(60.8)
**Education**		
Less than high school diploma		12(6.3)
High school diploma		30(15.9)
College or higher		147(77.8)
**Employment**		
Working full-time		110(58.2)
Working part-time		38(20.1)
Not working		41(21.7)
**Annual Income**		
Less than $10,000		41(21.7)
$10,000–19,999		30(15.9)
$20,000–39,999		64(33.9)
$40,000 or more		54(28.6)
**Place of Birth**		
United States (U.S.)		172(91.0)
Non-U.S.		17(9.0)
**Religion**		
Christian		149(78.8)
Non-Christian		40(21.2)
**Usual Place of Medical Care**		
Private doctor		121(64.0)
Health department/ER^a^		36(19.1)
Community clinic		32(16.9)
**Last HIV Test**		
Less than 6 months		151(79.9)
More than 6 months or never		38(20.1)
**Risk Behaviors**		
Received money, gifts, drugs for sex in the past 12 months		26(13.8)
Gave money, gifts or drugs for sex in the past 12 months		10(5.3)
STIs in past 12 months		24(12.7)
Condomless anal intercourse in past 6 months		
	Any Condomless RAI^b^	
	Yes	50(47.17)
	No	56(52.83)
	Any Condomless IAI^c^	
	Yes	55(47.83)
	No	60(52.17)
**Risk Score**		
1. 0		67(43.2)
2. 1		80(51.6)
3. 2		8(5.2)
**Expressed Willingness to Participate in HIV Vaccine Trials**		
Not willing		26(13.8)
Minimally willing		52(27.5)
Moderately willing		38(20.1)
Very willing		73(38.6)

^a^ ER: Emergency Room

^b^ RAI: Receptive anal intercourse

^c^ IAI: Insertive anal intercourse

79.9% (N = 151) of the participants reported having been tested for HIV within the last 6 months. A small proportion of the participants engaged in high-risk behaviors in the last 12 months, including receiving money, gifts, drugs for sex (N = 26; 13.8%); giving money, gifts, drugs for sex (N = 10; 5.3%); having been diagnosed with STIs in the past 12 months (N = 24; 12.7%). Furthermore, 47.2% (N = 50) and 47.8% (N = 55) of the participants reported engaging in condomless RAI and condomless IAI, respectively. Based on our analysis, 51.6% (N = 80) and 5.2% (N = 8) of the participants had a RS of 1 and 2, respectively. However, 43.2% (N = 67) had a RS of 0 (Table 1).

### HIV biomedical trials awareness and experiences

Almost three-quarter of the participants reported having heard of HIV vaccine trials (N = 141; 74.6%); only 8 (5.7%) of those ever participated in any of the HIV vaccine trials (Table 2). Six of those 8 reported good to excellent experience when enrolled in the trial. Only a small fraction (N = 18; 9.5%) reported knowing anyone who has participated in HIV vaccine trials. When assessing PrEP and PEP awareness, 60.8% (N = 113) had heard of PrEP, while 49.5% (N = 92) had heard of PEP. Of those reported having heard of PrEP and PEP, only 12.4% (N = 14) and 12% (N = 11) had ever used PrEP and PEP, respectively. Among PrEP users (N = 14), 10 (71.4%) had a RS > = 1. Among PEP users (N = 11), 10 (90.9%) had a RS > = 1. For former PrEP trial participants (N = 10), half reported having good to excellent experience, while half reported having only a fair experience.

**Table 2:** Knowledge and awareness of HIV vaccine and other biomedical prevention trials.

	Yes(%)
Ever heard of HIV vaccine trials (n = 184)	141(74.6)
Ever participated in any HIV vaccine trials (n = 141)	8(5.7)
Know anyone who has participated in HIV vaccine trials (n = 189)	18(9.5)
Ever heard of vaccine-induced seropositivity (n = 178)	36(20.2)
Ever heard of PEP (n = 186)	92(49.5)
Ever used PEP (n = 92)	11(12.0)
Ever heard of PrEP (n = 186)	113(60.8)
Ever used PrEP (n = 113)	14(12.4)
Ever heard of PrEP trials (n = 113)	28(24.8)
Ever participated in any PrEP trials (n = 113)	10(8.9)

### Factors associated with willingness to participate in HIV vaccine trials

In the bivariate analysis, we found significant association between WTP in HIV vaccine trials and demographic characteristics (education, employment, annual income, place of birth, religion, usual place of medical care) (Table 3). However, none of the risk behaviors, when calculated as a singular factor, were significantly associated with the outcome; only RS was found to be significant. Multivariable logistic regression analysis found that participants reported they would be less willing to participate in HIV vaccine trials (Table 3) if they had a Risk Score of 1 (OR = 0.39; p = 0.009) compared to a Risk Score of 0. We found no association between WTP in HIV vaccine trials and education, employment status, religion, income, place of birth, usual place of medical care, last HIV test, PrEP or PEP usage, and past HIV vaccine research participation (Table 3).

**Table 3:** Factors associated with Willingness to participate in HIV vaccine trials among those who have not participated in HIV vaccine trials in the past (N = 189) and multivariable logistic regression examining the association between willingness to participate in HIV vaccine trials and sociodemographic characteristics and risk score (N = 155).

		Not willing to participate (N = 78) N (%)	Willing to participate (N = 111) N (%)	P-value	OR (95% Cl)	P-value
**Education**				0.02		
Less than high school diploma		7(58.3)	5(41.7)		Reference	Reference
High school diploma		18(60.0)	12(40.0)		0.67(0.15–3.08)	0.61
College or higher		53(36.1)	94(63.9)		1.51(0.38–5.96)	0.56
**Employment**				0.001		
Working full-time		33(30.0)	77(70.0)			
Working part-time		24(63.2)	14(36.8)			
Not working		21(51.2)	20(48.8)			
**Annual Income**				<0.001		
Less than $10,000		21(51.2)	20(48.8)			
$10,000–19,999		21(70.0)	9(30.0)			
$20,000–39,999		21(32.8)	43(67.2)			
$40,000 or more		15(27.8)	39(72.2)			
**Place of Birth**				0.04		
United States		67(38.9)	105(61.1)		1.75(0.57–5.36)	0.33
Non-U.S.		11(64.7)	6(35.3)		Reference	Reference
**Religion**				0.007		
Christian		54(36.2)	95(63.8)			
Non-Christian		24(60.0)	16(40.0)			
**Usual Place of Medical Care**				0.02		
Private doctor		41(33.9)	80(66.1)			
Health department/ER^a^		21(58.3)	13(41.7)			
Community clinic		16(50.0)	16 (50.0)			
**Last HIV Test**				0.05		
Less than 6 months		57(37.8)	94(62.3)		1.16(0.52–2.60)	0.71
More than 6 months/never		21(55.3)	17(44.7)		Reference	Reference
**Risk Behaviors**						
Exchange money, gifts, drugs for sex in the past 12 months		13(50.0)	13(50.0)	0.33		
Gave money, gifts or drugs for sex in the past 12 months		6(60.0)	4(40.0)	0.32*		
STIs in past 12 months		13(54.2)	11(45.8)	0.17		
Condomless anal intercourse in past 6 months						
	Any condomless RAI^b^			0.85*		
	Yes	24(48.0)	26(52.0)			
	No	25(44.6)	31(55.4)			
	Any condomless IAI^c^			0.19*		
	Yes	31(56.4)	24(43.6)			
	No	26(43.3)	34(56.7)			
**Risk Score**				0.01		
1. 0		22(32.8)	45(67.2)		Reference	Reference
2. 1		45(56.2)	35(43.8)		0.39(0.19–0.79)	0.01
3. 2		5(62.5)	3(37.5)		0.37(0.08–1.84)	0.23
**PrEP**^d^ **or PEP**^e^ **Usage Ever In the Past**						
	PrEP			1.00		
	Yes	5(35.7)	9(64.3)			
	No	33(33.3)	66(66.7)			
	PEP			0.34*		
	Yes	4(36.4)	7(63.6)			
	No	45(55.6)	36(44.4)			
**Past Research Participation**						
	HIV vaccine trials			0.26*		
	Yes	1(12.5)	7(87.5)			
	No	48(36.1)	85(63.9)			

(*) = Fisher’s Exact Test

^a^ ER: Emergency Room

^b^ RAI: Receptive anal intercourse

^c^ IAI: Insertive anal intercourse

^d^ PrEP: Pre-Exposure Prophylaxis

^e^ PEP: Post-Exposure Prophylaxis

### Barriers and facilitators

Cash compensation, confidentiality regarding participation, public transportation vouchers, gift cards, and food or grocery vouchers were ranked highest as facilitators when considering participating in biomedical prevention trials (Table 4). The most common barriers were the following: fear of side effects from the vaccine, VISP, limited knowledge of research trials, and fear of being judged as HIV-positive. Interestingly, 37.6% (N = 71) reported that they had no barriers that would prevent them from participating in the HIV vaccine trials. Among those with no barriers, 90.1% (N = 64) had heard of HIV vaccine trials, but only 6.3% of these (N = 4) had ever participated in HIV vaccine trials (Data not shown).

**Table 4:** Barriers and facilitators to HIV vaccine trial participation (N = 189).

	N(%)
**Top 5 Facilitators**	
Cash compensation for my time	142(75.1)
Confidentiality regarding my participation	122(64.6)
Public transportation vouchers	121(64.0)
Gift cards	110(58.2)
Food or grocery vouchers	96(50.8)
**Top 5 Barriers**	
Fear of side effects from the vaccine	84(44.4)
Nothing	71(37.6)
Fear of testing false positive on routine HIV testing	57(28.0)
Limited knowledge of research trials	53(28.0)
Fear of being judged as HIV positive	43(22.8)

## Discussion

58.7% of the participants were willing to participate in HIV vaccine trials, which is comparable to previous studies conducted in young adults in Tanzania and MSM in India (50.6% and 48.1%, respectively) [13, 14], but lower than the 76.7% observed in China [15]. This number is slightly higher than the 46% reported in a study conducted in 3 U.S. cities. However, the population in the study was broadly recruited and included injecting drug users and MSM, while this study focused only on BMSM and TW [16].

Our results differed from those of past studies, which have shown high-risk MSM were more likely to expressed WTP in HIV vaccine trials than lower risk MSM. Our study included only young MSM and TW aged from 18–30 years, whereas the median age for the other studies was higher: participants in studies conducted by Newman et. al. at two sites in the U.S. had a median age of 33 and 37 years. Additionally, the study populations were both male and female, and did not exclusively focus on MSM and TW [15, 17, 18]. Another study with contradictory results was among the Thai Army who were male and aged between 21–29, suggesting higher willingness among high-risk individuals to participate in HIV vaccine trials when compared to those with lower risk [19]. Therefore, it is worth exploring whether high-risk young BMSM and TW face unique challenges, such as gender-affirming care settings, and a lack of legal protections for human rights, that deter them from engaging in trials [20, 21]. A prior study indicated that PrEP awareness was associated with previous history of STIs [22]; however, this association was not observed in our study.

We found that three-quarters of young BMSM and TW in our study were aware of HIV vaccine trials, but only 5.7% ever participated. This may be due to the fact that only 9.5% knew someone who had participated in the HIV vaccine trials, limiting referral among the participants and their social networks. Earlier studies noted the importance of familial and social support on HIV vaccine trials participation [14, 15].

Our study showed that cash compensation, confidentiality regarding participation, public transportation vouchers, gift cards, and food or grocery vouchers were top facilitating factors for participating in HIV vaccine trials. Our findings were similar to those found in studies conducted in China and Thailand, which had determined that financial incentives were associated with HIV vaccine participation [15, 19]. Since cash compensation was a significant factor for this population [15, 19], our result may indicate that young BMSM experienced financial insecurity. Conversely, we found that fear of side effects from the vaccine, false positive results on routine HIV testing, limited knowledge of research trials, and fear of being judged as HIV positive were perceived as barriers, which also echoed in other studies as major [23, 24]. This suggests that more education should be conducted to young BMSM and TW communities about HIV vaccine trials with full discussion of side effects and VISP. Coupled with what previous studies suggested regarding social support, it may also be beneficial if the studies are constructed based on a referral system, with financial incentives.

Another possible reason underlying low enrollment of BMSM and TW in research studies may be due to historical precedence, thus it may be valuable to explore social constructs around racial issues related to biomedical research. While it is important to educate and inform potential research participants, it is imperative to provide social and psychological support as previous studies suggested that these are important components that are highly correlated with WTP [14, 15]. A recent HPTN 073 showed that a theory-based, culturally tailored programs for Black MSM, such as counseling, led to high uptake (79% at week 0 and 68% at 26 weeks) as well as high adherence (85% at 4 weeks and 78% at 26 weeks) to PrEP [25].

This study has several limitations. There is a lack of generalizability to BMSM and TW in other metropolitan or rural areas, as well as races, ethnicities, and age. Additionally, we did not stratify BMSM and TW for our analyses, thus we could not capture specific risks and results for each group. Moreover, the WTP is based on a hypothetical setting, thus the findings may not accurately apply to actual trials. [26] Furthermore, due to the small sample size, the power of the results is limited. Also, the data may be subject to self-reporting bias, though the online survey system should limit this bias.

Nevertheless, our findings revealed factors that could enhance enrollment of young BMSM and TW in HIV vaccine trials. Future studies should investigate factors such as cultural-societal barriers, and ways to expand study participation through social support and peer referrals. Furthermore, qualitative studies are important to understand complex mechanisms that online questionnaire are unable to capture, which could contribute to or contradict our findings with regard to why lower risk individuals were more willing to participate in HIV vaccine trials than their high-risk counterparts.

## Conclusion

Young Black MSM and transwomen have been disproportionately burdened by the HIV epidemic, and the generalizability from the results of biomedical prevention studies may be compromised due to the lack of their enrollment. This study examined the key factors as well as risk characteristics that influenced young Black MSM and TW in NYC on HIV vaccine trial participation, aiming to find potential ways to enhance the involvement of Black MSM and TW community in HIV vaccine trials. We found that low-risk persons were much more likely to express willingness to participate in HIV vaccine trials when compared to high-risk persons, and our results also indicated that financial incentives could potentially increase the enrollment in in such studies. Caution may be advised if monetary reimbursement serves as an undue influence. Additionally, more efforts should be invested in creating referral system and supportive environment to increase the engagement of Black MSM and transwomen in HIV vaccine research trials.

## Supporting information

S1 File Prescreener questionnaire. (PDF)

S2 File Main survey. (PDF)
