# Supplementary material for: Barriers and facilitators of HIV vaccine and prevention study participation among Young Black MSM and transwomen in New York City
Source: PLoS One. 2017 Jul 19;12(7):e0181702. doi: 10.1371/journal.pone.0181702 (PMC5517061; doi:10.1371/journal.pone.0181702)
Supplement: S1 File — (PDF) [file pone.0181702.s001.pdf]

Prescreener Questionnaire:

Introduction:

The New York Blood Center is doing a research study to learn more about what men think about studies that try to find new ways to prevent HIV infection. If you are eligible and decide to take part, you will receive a $30 gift card for answering an on-line survey (lasting about 20-30 minutes). Click "next page" to answer 8 questions to see if you are eligible.

1. What is your age?

[If under age 18 or over 30 years old…] *I'm sorry but you are not eligible to participate in this study at this time. Thank you for your interest.*

2. What is your gender?
   - ☐ Male
   - ☐ Female→ (ineligible)
   - ☐ Transgender
     - ☐ Male to female
     - ☐ Female to male (ineligible)

3. What is your race/ethnicity (check all that apply)?

☐ Black/Non-Hispanic or African American

☐ White/Non-Hispanic

☐ Hispanic/Latina

☐ Asian/Pacific Islander

☐ Native American/Alaskan Native

☐ Mixed Race, specify ______________________________

☐ Other, specify__________________________________

4. Do you live in the New York City area, including the 5 boroughs?

   ☐ No (Ineligible)

   ☐ Yes

5. In the past **6 months**, have you had vaginal or anal sex with a woman?

- ☐ No
- ☐ Yes

6. In the past **6 months**, have you had anal sex with a man?

- ☐ No (ineligible)
- ☐ Yes

8. What were the results of your last HIV test?

- ☐ Never tested/unknown
- ☐ Negative
- ☐ Positive (Ineligible)

Eligible for study:

- ☐ **Yes**
- ☐ **No**

If eligible:

Thank you for answering these questions. You are eligible to participate in the study. You will receive a $30 gift card when you complete the study survey, which will take about 20-30 minutes. Are you interested in taking part?

Yes (click will take participant to survey)

No (Thank you for your time. If you would like to learn more about our other studies, please call Project Achieve at 212-388-0008 or 718-402-0743).

For those who are ineligible:

“I’m sorry but you are not eligible to take part in this study. There are many reasons why people are not eligible to participate, reasons that were decided earlier by the researchers. We appreciate your interest in participating and willingness to consider helping our community.. If you would like to learn more about our other studies, please call Project Achieve at 212-388-0008 or 718-402-0743.
