# Supplementary material for: Barriers and facilitators of HIV vaccine and prevention study participation among Young Black MSM and transwomen in New York City
Source: PLoS One. 2017 Jul 19;12(7):e0181702. doi: 10.1371/journal.pone.0181702 (PMC5517061; doi:10.1371/journal.pone.0181702)
Supplement: S2 File — (PDF) [file pone.0181702.s002.pdf]

Main Survey:

Introduction:

Thank you for joining this study. This survey will ask you many questions on a number of topics. Topics will include your education level, income, employment status, participation in previous general medical research studies and previous HIV prevention and vaccine trials. We are also interested in your knowledge, perceptions of, and willingness to participate in HIV vaccine and biomedical prevention intervention research. No information that can be traced back to you will be collected. You will receive a $30 gift card after finishing for your participation.

Thank you again for agreeing to participate in the survey.

Demographics

D1. What is your highest level of education?

1 Did not complete high school
2 Completed vocational/technical/trade school, without high school diploma or GED
3 Completed vocational/technical/trade school with high school diploma or GED
4 Completed high school or GED without vocational/technical/trade school
5 Some college or two year degree
6 Completed college (4 years)
7 Graduate school or higher

D2. Are you currently enrolled in school?

0 No
1 Yes, full time
2 Yes, part time

D3. What best describes your current employment status? (Choose one)

1 Working full-time
2 Working part-time (including seasonal/work-study etc.)
3 Not working, but actively looking for work
4 Not working, not actively looking for work
5 Temporarily laid off

6 Retired
7 Working off the books (for example, recycling cans/bottles, selling drugs or sex, panhandling)
8 Other

D4. What is your total annual personal income?

1 Less than $10,000
2 $10,000-19,999
3 $20,000 - 39,999
4 $40,000 - 59,999
5 $60,000 – 79,999
6 $80,000 – 99,999
7 $100,000 – 119,9998
8. $120,000+

D5. Do you have health insurance?

1 Yes
2 No
3 I don’t know

D6. What is your ethnic background?

1 American
2 West-Indian
3 African
4 Hispanic

D7. What is your place of birth?

1 United States
2 The Caribbean
3 South America
4 Central America
5 Africa
6 Asia
7 Europe
8 Canada
9 Australia, Oceania
10 Other

D8. Where is your religious affiliation?

1 Christian
2 Jewish

3 Muslim
4 Hindu
5 Buddist
6 Atheist
7 Other

D9. Where do you usually go for medical care?

1 I have a private doctor
2 Health department
3 Emergency room
4 Community clinic
5 I do not seek medical care

D10. When was the last time you were tested for HIV?

1 This past month
2 Within the last 3 months
3 Within the last 4- 6 months
4 Within the last 7-12 months
5 More than one year ago
6 Never

Knowledge

K1. Have you ever heard of HIV vaccine trials?

1 Yes **(Skip to K1.1 and K1.2)**
2 No
3 I don't know

K1.1. (If yes to the question above) How would you rate your knowledge/familiarity in regards to HIV vaccine trials (from 1 to 4)

1 Not much (example: Only having knowledge of the name)
2 Limited (Example: I know there is research but am not aware of where it stands)
3 Moderate (example: I know about past trial results, where current trials are being held, and I know the current status of research)
4 Expert (Example: I know very well about past trial results, where and what current trials are held, and I am well read when it comes to HIV prevention research)

K1.2. (If answered yes to the question K1) Do you know of any HIV vaccine trials going on in your area?

1 Yes
2 No

K2. Have you ever participated in any HIV vaccine trials?

1 Yes **(Skip to K2.1 and K2.2)**
2 No

K2.1.(If yes), Are you now participating in any HIV vaccine trials?

1. Yes
2. No

K2.2 (If yes to the questions K2 or K2.1 above), Please rate your overall experience of participation in an HIV vaccine trial:

1. Excellent
2. Good
3. Fair
4. Poor

K3. Do you know anyone who has participated in HIV vaccine trials?

1 Yes
2 No

K4. Have you ever heard of vaccine-induced seropositivity (testing HIV positive on a routine HIV test due to getting an HIV vaccine but not actually having HIV)?

1 Yes
2 No
3 I don't know

K5. Have you ever heard of any HIV post-exposure prophylaxis (PEP) (HIV medications you take within a few days of getting exposed, either sexually or by needle, to someone with HIV infection)?

1 Yes
2 No
3 I don't know

K6. Have you ever used HIV post-exposure prophylaxis (PEP)? (HIV medications you took within a few days of getting exposed, either sexually or by needle, to someone with HIV infection)?

1 Yes
2 No

K6.1 (If yes to K6) On a scale of 1 to 5, please rate your level of adherence to post-exposure prophylaxis protocol.

1 I did not take any medications or recommendations
2 I took very few medications and/or followed few recommendations
3 I took most medications and/or followed most recommendations
4 I took almost all medications and/or followed almost all recommendations
5 I took all medications and followed all recommendations

K7. Have you ever heard of HIV pre-exposure prophylaxis (PrEP)? (HIV medications you take every day to prevent you from getting infected with HIV)

1 Yes
2 No
3 I don't know

K8. Have you ever used HIV pre-exposure prophylaxis (PrEP)?

1 Yes (Skip to K8.1)
2 No

K8.1. (If yes to K8) On a scale of 1 to 5, please rate your level of adherence to pre-exposure prophylaxis (PrEP)

1 I did not take any medications or recommendations
2 I took very few medications and/or followed few recommendations
3 I took most medications and/or followed most recommendations
4 I took almost all medications and/or followed almost all recommendations
5 I took all medications and followed all recommendations

K9. Do you know of any HIV pre-exposure prophylaxis (PrEP) trials?

1 Yes
2 No

K10. Have you ever participated in any HIV pre-exposure prophylaxis (PrEP) trials?

1 Yes
2 No

K10.1 (If yes to the questions K10 above), Please rate your overall experience of participation in an HIV pre-exposure prophylaxis (PrEP) trial:

1 Excellent
2 Good
3 Fair
4 Poor

K11. Have you ever heard of HIV rectal microbicide research trials (special gel you put in the rectum to prevent you from getting HIV)?

1 Yes
2 No

Willingness to participate

W1. On a scale from 1 to 4 with 1 being not willing and 4 being very willing, how willing are you to participate in an HIV vaccine trial if invited today?

1 Not willing
2 Minimally willing
3 Moderately willing
4 Very willing

W2. On a scale from 1 to 4 with 1 being not willing and 4 being very willing, how willing are you to participate in an HIV pre-exposure prophylaxis (PrEP) trial if invited today?

1 Not willing
2 Minimally willing
3 Moderately willing
4 Very willing

W2.1 On a scale from 1 to 4 with 1 being not willing and 4 being very willing, how willing are you to participate in an HIV pre-exposure prophylaxis (PrEP) trial that involves an **injection** with a needle if invited today?

1 Not willing
2 Minimally willing
3 Moderately willing
4 Very willing

W2.3 On a scale from 1 to 4 with 1 being not willing and 4 being very willing, how willing are you to participate in an HIV pre-exposure prophylaxis (PrEP) trial that involves **a medication you take by mouth** if invited today?

1 Not willing
2 Minimally willing
3 Moderately willing
4 Very willing

W3. On a scale of 1 to 5, with 1 being least important and 5 being the most important, please rate the importance of the following items in terms of what would make your participation in a research trial easier or harder:

1 Private transportation to and from appointments ____
2 Public transportation (bus, metro) vouchers ____
3 Cash compensation for your time ____
4 Food or grocery vouchers ____
5 Gift cards ____
6 Referrals for other community services ____
7 Condoms, dental dams
8 Hygiene kits (toothbrush, toothpaste, bleach, soaps, shampoo, etc.) ____
9 Clothing ____
10 Weekday morning study visits ____
11 Weekday evening study visits ____
12 Weekend study visits ____
13 Going to a site close to my home ____
14 Confidentiality regarding my participation ____
15 Other commitments (work, family, etc.)
16 Child care or reimbursement for cost of child care ____

W4. On a scale of 1 to 5, with 1 being least important and 5 being the most important, please rate the importance of the following items on whether or not you would participating in a research trial:

1 Participating in a trial helps my community ____
2 Participating in a trial helps other men who have sex with men ____
3 Participating in a trial potentially helps me stay HIV-negative ____
4 Participating in a trial provides a means for evaluating my health on an ongoing basis__
5 Participating in a trial makes me eligible for incentives ____
6 Participating in a trial gives me access to ongoing risk reduction counseling ___
7 Participating in a trial makes me feel like I matter ____
8 Participating in a trial makes me feel worthy ____
9 Participating in a trial makes me eligible to receive compensation ____

W5. Would you invite friends and/or family members to participate in an HIV vaccine trial?

1 Yes
2 No
3 I don't know

W6. (If No or I don't know), Please put the following items in order of importance for your decision to not invite friends and/or family members to an HIV vaccine trial, with 1 being most important and 7 being the least important.

1 Previous negative experience in trial
2 Distrust of researchers
3 Vaccine has not be tested enough
4 Fear that some may assume you are infected with HIV
5 Lack of incentives
6 Do not have friends and/or family who would qualify for trial
7 Do not have friends and/or family that are at risk for contracting HIV

W7. Would you invite friends and/or family members to participate in an HIV pre-exposure prophylaxis (PrEP) research trial that involves taking pills by mouth?

1 Yes
2 No
3 I don’t know

W8. Would you invite friends and/or family members to participate in an HIV pre-exposure prophylaxis (PrEP) research trial that involves injection with a needle?

1 Yes
2 No
3 I don’t know

W9. Would you invite friends and/or family members to participate in an HIV rectal microbicide research trial (special gel you put in the rectum to prevent HIV infection)

1 Yes
2 No
3 I don’t know

Barriers

B1. What would prevent you from participating in an HIV vaccine trial? (mark all that apply)

1 Access to study site
2 Fear of being judged as HIV positive
3 Fear of being judged of being at risk for HIV infection
4 Fear of being judged of being gay
5 Fear of testing false positive on routine HIV testing
6 Limited knowledge of research trials
7 Fear of needles and injections
8 Fear of side effects from the vaccine
9 Fear of being discriminated against because of my race
10 Fear of being discriminated against because of my age
11 Nothing. I would be willing to participate

B1.1 What would prevent you from participating in an HIV pre-exposure prophylaxis (PrEP) research trial? (mark all that apply)

1 Access to study site
2 Fear of being judged as HIV positive
3 Fear of being judged of being at risk for HIV infection
4 Fear of being judged of being gay
5 Fear of not being able to take medication on a regular basis as prescribed
6 Limited knowledge of research trials
7 Fear of needles and injections (for PrEP studies involving injections)
8 Fear of side effects from the medication
9 Fear of being discriminated against because of my race
10 Fear of being discriminated against because of my age
11 Nothing. I would be willing to participate

B1.2 What would prevent you from participating in an HIV rectal microbicide research trial? (mark all that apply)

1 Access to study site
2 Fear of being judged as HIV positive
3 Fear of being judged of being at risk for HIV infection
4 Fear of being judged of being gay
5 Fear of not being able to use the rectal gel on a regular basis as prescribed
6 Limited knowledge of research trials
7 Fear of putting a gel in my rectum
8 Fear of side effects from the medication
9 Fear of being discriminated against because of my race
10 Fear of being discriminated against because of my age
11 Nothing. I would be willing to participate

B2. Have you ever chosen to not participate in other forms of clinical research in the past?

1. Yes
2. No

B3. Have you ever been invited to participate in HIV vaccine trials?

1 Yes
2 No
3 I don't know

B3.1. (If yes to B3) Did you participate in the trial?

1 Yes, I participated
2 No, I did not participate

B3.2. (If yes answered to the question above) Please mark all the factors below that influenced your decision

1 Did not trust those administering the trial
2 The location was not convenient
3 I did not have time
4 Other participants were not of similar race, sexual, and/or income background
5 Uncomfortable with subjecting myself to research

B4. Have you ever been invited to participate in an HIV pre-exposure prophylaxis (PrEP) research trial?

4 Yes
5 No
6 I don't know

B4.1. (If yes to B4) Did you participate in the trial?

3 Yes, I participated
4 No, I did not participated

B4.2. (If yes answered to the question above) Please mark all the factors below that influenced your decision

6 Did not trust those administering the trial
7 The location was not convenient
8 I did not have time
9 Other participants were not of similar race, sexual, and/or income background
10 Uncomfortable with subjecting myself to research

Perceptions

P1. On a scale from 1 to 5, with 1 being not important and 5 being very important, how important do you think it is that a cure is found for HIV?

1 Not important
2 Somewhat important
3 Minimally important
4 Moderately important
5 Very important

P2.On a scale from 1 to 5, with 1 being not worried and 5 being extremely worried and 1 being not worried at all, how worried are you about being infected with HIV?

1 Not worried
2 Minimally worried
3 Moderately worried
4 Very worried
5 Extremely worried

P3. If a vaccine for HIV is found to be effective in preventing HIV infection and is approved for use, would you get it?

1 Yes
2 No
3 I don't know

P3.1 (If answered No or I don't know to P3) please mark all of the factors below that influence your opinion

1 Distrust of health providers
2 Vaccine has not been fully tested
3 Vaccine has not been fully tested in young black men who have sex with men

4 Lack of incentives
5 I am not at risk for HIV and therefore do not need a vaccine
6 I will not have money to pay for vaccine
7 I am afraid of needles and don't like injections
8 I am afraid of side effects from the vaccine

P4. If an HIV pre-exposure prophylaxis (PrEP) drug that requires injection with a needle is found to be effective in preventing HIV infection and is approved for use, would you use it?

1 Yes
2 No
3 I don't know

P5. If an HIV rectal microbicide is found to be effective in preventing HIV infection and is approved for use, would you use it?

1 Yes

2 No

3 I don't know

Sexual Behaviors

S1. In the last **12 months**, did you receive money, drugs, gifts or services in exchange for sex?

1 No

2 Yes

S2. In the last **12 months**:

S2.1 did you have a sexually transmitted infection or STD (for example gonorrhea, Chlamydia, syphilis)?

1 No
2 Yes

S2.2 did you inject drugs?

1 No
2 Yes

S2.3 did you use cocaine?

1 No
2 Yes

S2.4 did you use methamphetamine/amphetamine? [crystal meth]

1 No
2 Yes

S2.5 did you use any other drugs?

1 Yes
2 No
3 I don't know

S3. In the past **6 months**, how many men have you had anal sex (top or bottom) with?

1 One
2 Two
3 Three
4 Four or more___

S4. In the past 6 months, how many men have you had anal sex (top or bottom) with and used a condom?

1 None
2 One
3 Two
4 Three
5 Four or more___

S5. In the past 6 months, how many men have you had anal sex (top or bottom) with and did NOT use a condom?

1 None
2 One
3 Two
4 Three
5 Four or more___

S6. In the **past 6 months**, how many men have you had oral sex with? ____ (specify number)

S7. Have you ever been vaccinated against Hepatitis (A and/or B)?

1 Yes
2 No
3 I don't know

This is the end of the survey. Thank you for your help. Your participation is very much appreciated.
